# Structure of the GH76 α-mannanase homolog, BT2949, from the gut symbiont *Bacteroides thetaiotaomicron*

**DOI:** 10.1107/S1399004714026443

**Published:** 2015-01-23

**Authors:** Andrew J. Thompson, Fiona Cuskin, Richard J. Spears, Jerome Dabin, Johan P. Turkenburg, Harry J. Gilbert, Gideon J. Davies

**Affiliations:** a Department of Chemistry, University of York, Heslington, York YO10 5DD, England; b Institute for Cell and Molecular Biosciences, Newcastle University, Newcastle upon Tyne NE2 4HH, England

**Keywords:** GH76, BT2949, *Bacteroides thetaiotaomicron*

## Abstract

The large bowel microbiota, a complex ecosystem resident within the gastrointestinal tract of all human beings and large mammals, functions as an essential, nonsomatic metabolic organ, hydrolysing complex dietary polysaccharides and modulating the host immune system to adequately tolerate ingested antigens. A significant member of this community, *Bacteroides thetaiotaomicron*, has evolved a complex system for sensing and processing a wide variety of natural glycoproducts in such a way as to provide maximum benefit to itself, the wider microbial community and the host. The immense ability of *B. thetaiotaomicron* as a ‘glycan specialist’ resides in its enormous array of carbohydrate-active enzymes, many of which are arranged into polysaccharide-utilization loci (PULs) that are able to degrade sugar polymers that are often inaccessible to other gut residents, notably α-mannan. The *B. thetaiotaomicron* genome encodes ten putative α-mannanases spread across various PULs; however, little is known about the activity of these enzymes or the wider implications of α-mannan metabolism for the health of both the microbiota and the host. In this study, SAD phasing of a selenomethionine derivative has been used to investigate the structure of one such *B. thetaiotaomicron* enzyme, BT2949, which belongs to the GH76 family of α-mannanases. BT2949 presents a classical (α/α)_6_-barrel structure comprising a large extended surface cleft common to other GH76 family members. Analysis of the structure in conjunction with sequence alignments reveals the likely location of the catalytic active site of this noncanonical GH76.

## Abstract 2

A high-resolution structure of a noncanonical α-mannanase relevant to human health and nutrition has been solved *via* heavy-atom phasing of a selenomethionine derivative.

## 1. Introduction   

**Figure 1:**
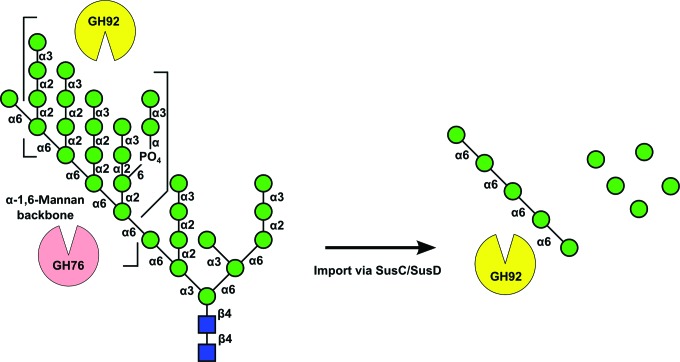
The structure of *S. cerevisiae* α-mannan and a potential model for its hydrolysis by *Bt*. Exo-acting GH92 α-­mannosidases remove α-1,2- and α-1,3-linked side-chain moieties, whilst GH76 enzymes hydrolyse the α-­1,6 backbone, allowing the import of smaller mono-oligosaccharides for further digestion. Carbohydrate residues are shown according to CFG (Centre for Functional Glycomics) nomenclature: green circles denote mannose, whilst blue squares represent *N*-acetylglucosamine. Linkages are specified.

*Bacteroides thetaiotaomicron* (*Bt*), a dominant member of the microbiota, the symbiotic microbial community that inhabits the large bowel of humans and other higher mammals, plays an essential role in health and nutrition through its modulation of otherwise intractable dietary polysaccharides (Xu & Gordon, 2003 ▶; Bäckhed *et al.*, 2005 ▶). Indeed, *Bt* represents one of the largest expansions of carbohydrate-processing enzymes known, with approximately 10% of its protein-encoding genes devoted solely to the production of catabolic glycoside hydrolase (GH) and anabolic glycosyltransferase (GT) enzymes (Xu *et al.*, 2003 ▶). Of the various GHs encoded by *Bt*, a subset of over 30 enzymes are predicted to specifically target α-mannosidic linkages through their assignment to known α-mannosidase/α-mannanase families within the sequence-based CAZy classification (Lombard *et al.*, 2014 ▶; http://www.cazy.org). The evolution of such a large catalytic repertoire to target a glycosidic bond observed only relatively scarcely in nature therefore suggests α-mannose-containing polysaccharides, such as the α-mannan present in yeast cell walls, to be a significant nutrient for both *Bt* and other closely related gut-dwelling bacteria. However, given their apparently low biological abundance (compared with, for example, β-mannose- or glucose-containing polysaccharides) and recalcitrant chemistry (nucleophilic substitution at the anomeric centre of mannosides is among the most difficult known; reviewed in Crich, 2010 ▶; Davies *et al.*, 2011 ▶), the deliberate selection of α-mannosides as a primary carbon source therefore seems to be quite unusual. One likely line of reasoning for this utilization can be found in the continued evolution of the host diet. Over the last several thousand years, human beings have increasingly taken advantage of microorganism-catalysed fermentation reactions in the production of foods and beverages, such as bread and beer, a process exemplified by the now common referral to the budding yeast *Saccharo­myces cerevisiae* as so-called ‘brewer’s or baker’s yeast’. As such, consumption of yeast extracts, particularly α-mannan (for the structure of *S. cerevisiae* α-mannan, see Fig. 1 ▶), is now commonplace, strongly increasing their prevalence within the gut environment, and together with the highly competitive nature of the microbiota provides a strong evolutionary driving force towards the adaption of enzymes capable of processing this increasingly abundant and less commonly employed nutrient (Walter & Ley, 2011 ▶; Koropatkin *et al.*, 2012 ▶).

The microbiota represents one of the most densely populated and indeed well studied microbial communities known (Xu *et al.*, 2003 ▶; Koropatkin *et al.*, 2012 ▶). Comprising an estimated 10^14^ bacterial cells (well in excess of somatic host cells) and made up of hundreds of different species (Hooper & Gordon, 2001 ▶), both the composition and general health of the microbiota has been shown to have profound effects upon the health of the host (Turnbaugh *et al.*, 2006 ▶), not only through the ability to access otherwise challenging polysaccharides but also through the capacity of symbionts, such as *Bt*, to modulate the immune system to accommodate varying bacterial as well as dietary and environmental antigens (Mazmanian *et al.*, 2008 ▶; Wen *et al.*, 2008 ▶; Hooper & Macpherson, 2010 ▶). Furthermore, the specific ability of *Bt* to process yeast cell-wall components may well confer an ability to regulate the balance and spread of potentially pathogenic fungal communities (Xu *et al.*, 2007 ▶). As such, great research effort has been focused upon investigating factors that govern the normal composition and healthy metabolic functioning of the microbiota, with the aim of developing dietary strategies or supplements through which humans may directly regulate these internal ecosystems. External treatment strategies that intentionally influence the configuration or the performance of the microbiota would be strongly augmented by detailed study of the environmental response to various dietary glycans or glycoconjugates and biochemical characterization of the enzymes associated with their metabolism.

The majority of α-mannan-active genes encoded by the *Bt* genome are assigned to families GH92 and GH76 (23 and ten entries, respectively) within the CAZy database (Lombard *et al.*, 2014 ▶). As has been shown previously, GH92 comprises a family of metal-dependent exo-α-mannosidases with the major enzymatic activities targeting α-1,2 and α-1,3 glycosidic bonds, whilst a smaller number are specific for α-1,4-linked and α-1,6-linked mannosides (Zhu *et al.*, 2010 ▶). By comparison, early studies have shown GH76 enzymes to be far more specific, with activity only detectable against the extended α-1,6-mannoside backbone present in yeast α-mannans (Nakajima *et al.*, 1976 ▶). Together, these observations suggest a potential model for α-mannopolysaccharide breakdown whereby exo-acting enzymes, such as GH92 mannosidases, catalyse the removal of terminal α-1,2-mannosides and α-1,3-mannosides from the branches of yeast α-mannan (and potentially host N-glycans), whilst GH76 enzymes act to internally cleave the α-1,6-mannan backbone, thus forming smaller oligosaccharides for digestion by exo-α-1,6-mannosidases (Fig. 1 ▶). Such a potential blueprint for activity is lent support through the observation that genes encoding GH76 and GH92 enzymes often appear clustered together in various polysaccharide-utilization loci (PULs) throughout the *Bt* genome (Bjursell *et al.*, 2006 ▶; Martens *et al.*, 2008 ▶, 2009 ▶), an arrangement not dissimilar to that recently reported for the breakdown of xyloglucan by the closely related bacterium *B. ovatus* (*Bo*; Larsbrink *et al.*, 2014 ▶). Four GH76 structures have been released to date, with biochemical analysis of a small number of enzymes in the press (Cuskin *et al.*, 2015 ▶). Much work still remains to be performed in understanding the role that this family plays in mannose foraging by *Bt* and the wider implications for the general health and functioning of the microbiota.

In this paper, we present the crystal structure, determined by SAD phasing of a selenomethionine derivative, of one such GH76 enzyme from *Bt*, BT2949. Furthermore, we show that despite poor homology at the primary amino-acid level, the tertiary structure of BT2949 appears to be well conserved in other GH76 family members. These enzymes form a long trough-like structure, which is consistent with a requirement to accommodate extended polysaccharides such as α-mannan. Structural analysis and mapping of sequence conservation has allowed putative assignment of the location of the catalytic active site, which is shown to be different to the canonical site observed previously in this family, whilst a potential role in mannan breakdown is also discussed.

## 2. Experimental   

### 2.1. Cloning, expression and purification   

BT2949 was amplified by PCR using the primers BT2949FWD, 5′-CAACACGCTAGCGGCTGTGATGCCA­CTGTACAGGATATC-3′, and BT2949REV, 5′-CAACACC­TCGAGTTAATCGTTGAGTATTCTTTTGTAATT-3′. The amplified gene was cloned into pre-digested pET-28a (N-terminal His_6_ tag) using NheI and XhoI restriction sites.

*Escherichia coli* BL21 cells harbouring pET-28a-BT2949 were cultured in 0.5 l lysogeny broth (LB) supplemented with 50 µg ml^−1^ kanamycin at 310 K until the mid-exponential phase was reached (OD_600 nm_ of ∼0.8). Recombinant gene expression was induced by the addition of 0.2 m*M* (final concentration) isopropyl β-d-1-thiogalactopyranoside (IPTG) and further incubation at 290 K for approximately 16 h. A selenomethionine-labelled derivative of BT2949 was produced by incubating *E. coli* BL21 cells harbouring the plasmid in 0.5 l PASM5052 autoinduction medium (Studier, 2005 ▶) supplemented with 50 µg ml^−1^ kanamycin at 310 K for 8 h, with induction occurring during overnight incubation at 290 K. In all cases, the cells were harvested, resuspended in 50 m*M* HEPES pH 7.0, 300 m*M* NaCl and lysed by sonication.

Soluble lysates of both native and derivatized BT2949 were applied onto an NiSO_4_-charged 5 ml HiTrap chelating column (GE Healthcare) pre-equilibrated in the same buffer. The protein was eluted in an imidazole gradient, dialyzed, concentrated and further purified on a Superdex 75 16/60 gel-filtration column (GE Healthcare) pre-equilibrated in 25 m*M* HEPES pH 7.0, 100 m*M* NaCl. Pure protein-containing fractions were pooled and concentrated to approximately 40 mg ml^−1^ prior to crystallization screening.

### 2.2. Crystallization, data collection and structure solution   

Native BT2949 was screened for crystallization at a concentration of approximately 20 mg ml^−1^, with preliminary hits obtained in several conditions from Crystal Screen and Crystal Screen 2 (Hampton Research, Aliso Viejo, California, USA). Selenomethionine-derivatized BT2949 was screened at a concentration of approximately 40 mg ml^−1^ following a 30 min incubation with 5 m*M* tris(2-carboxyethyl)phosphine (TCEP). Hits were obtained in both the PACT premier (Molecular Dimensions, Newmarket, England) and Index screens (Hampton Research). Native BT2949 crystals were subsequently grown by hanging-drop vapour diffusion at 292 K in both 1:1 and 2:1 ratios of protein solution:reservoir solution, with the reservoir solution consisting of 100 m*M* Tris pH 7.0, 1.25 *M* ammonium phosphate. Crystals of the selenomethionine derivative were grown under identical conditions with a reservoir solution consisting of 1.5 *M* ammonium tartrate pH 6.6, 5 m*M* TCEP. All crystals were cryoprotected in the respective reservoir solutions supplemented with either (final concentrations) 20%(*w*/*v*) glycerol (native crystals) or 25%(*w*/*v*) ethylene glycol (derivative crystals). Diffraction data for both native crystals and selenomethionine-derivative BT2949 crystals were collected on beamline I03 at the Diamond Light Source (Didcot, Oxfordshire, England). Measured reflection intensities for all data sets were indexed, integrated and scaled using *XDS* (Kabsch, 2010*a* ▶,*b* ▶) and the *CCP*4 suite (Winn *et al.*, 2011 ▶) implementation of *AIMLESS*. The structure of BT2949 was solved by single-wavelength anomalous dispersion (SAD) phasing of the selenomethionine-derivative data set at a peak absorption energy of 12.661 keV (λ = 0.9793 Å). Selenium site location, refinement of heavy-atom positions and density modification were carried out using the *SHELXC*/*D*/*E* automated pipeline (Sheldrick, 2010 ▶), with 17 sites located at occupancies of 0.5 or greater (FOM at 2.5 Å = 0.76). An initial atomic model of SeMet-BT2949 was constructed using the *CCP*4 implementation of *Buccaneer* (Winn *et al.*, 2011 ▶) and was refined to convergence *via* the maximum-likelihood method using *REFMAC* (Murshudov *et al.*, 2011 ▶; Winn *et al.*, 2011 ▶), with additional manual correction using *Coot* (Emsley *et al.*, 2010 ▶). A high-resolution native data set was solved by molecular replacement with *MOLREP* (Winn *et al.*, 2011 ▶), using the selenomethionine-derivative coordinates as a phasing model. Native BT2949 was refined and corrected as detailed above. Models and structure factors for both native and derivative BT2949 have been deposited in the PDB with accession codes 4v1s and 4v1r, respectively. Final data-processing and refinement statistics are detailed in Table 1 ▶.

## 3. Results and discussion   

**Figure 2:**
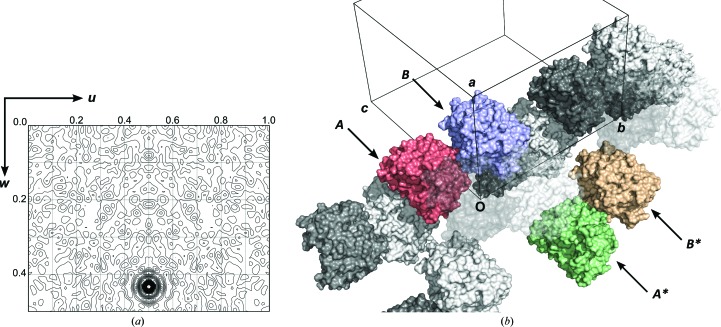
(*a*) BT2949 native Patterson map, *v* = 0.5 section, showing a large non-origin peak at *w* = 0.43 (peak heights are depicted relative to the origin). (*b*) Arrangement of molecules along the crystallographic *b* axis within the structure of BT2949. The two molecules that make up the asymmetric unit are labelled *A* and *B* (red and blue). Molecule *A* is related to molecule B* (yellow) by a translation of (½*x*, ½*y*, 0.43*z*). (*a*) was prepared using *FFT*/*MapSlicer* within the *CCP*4 suite (Winn *et al.*, 2011 ▶). (*b*) was assembled using *PyMOL* v.1.6 (Schrödinger).

The crystals of BT2949 belonged to the orthorhombic space group *P*22_1_2_1_ (unit-cell parameters *a* = 81, *b* = 121, c = 126 Å, α = β = γ = 90°) with two protein molecules present in each asymmetric unit (molecules *A* and *B* appear identical, with an average r.m.s.d of 0.07 Å across matching C^α^ positions). The final atomic model for each monomer spans a continuous peptide chain comprising residues Pro37–Asn395 (residues 1–22 form a signal peptide and were removed in this construct to allow soluble gene expression). Initial merging of the data with *AIMLESS* in space group *P*2_1_2_1_2_1_ appeared satisfactory (systematic absence probability of ≥0.98 for three 2_1_ screw axes); however, the structure could not be solved after several attempts at SAD phasing, with poor contrast and mapCC (correlation coefficient) between the two possible hands for the structure. Closer inspection of diffraction intensities and the native Patterson (Fig. 2 ▶ *a*) revealed likely translational noncrystallographic symmetry (tNCS). Subsequent reprocessing of the data in *P*22_1_2_1_ allowed facile phasing of the selenium substructure and model building of two complete BT2949 molecules. Analysis of the refined structure revealed the observed tNCS to be owing to two identically orientated molecules in neighbouring asymmetric units related to the origin by a translation of (½*x*, ½*y*, 0.43*z*) (see Fig. 2 ▶ *b*).

**Figure 3:**
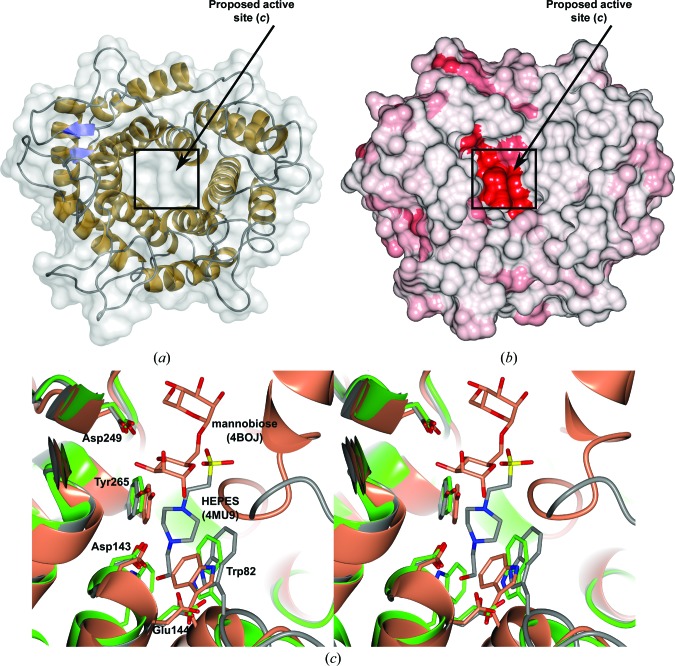
(*a*) The single-domain (α/α)_6_ fold of BT2949 viewed along the barrel axis. The barrel appears relatively undecorated, with only four major loops (shown in grey) comprising two small elements of secondary structure (shown in blue). (*b*) Surface view of BT2949 [identical orientation to that in (*a*)] coloured by sequence conservation using the alignment shown in Fig. 4 ▶(*a*); colour intensity indicates strongest conservation. The position of the proposed catalytic active site in (*a*) and (*b*) is indicated *via* an arrow and the view shown in (*c*) *via* a boxed region. (*c*) Divergent stereo image showing HEPES bound in BT3782 (PDB entry 4mu9, coloured grey) and α-1,6-mannobiose in *Bc*GH76 (PDB entry 4boj, coloured coral) overlaid with BT2949 (green). HEPES makes a hydrogen-bonding interaction with Asp143 (all labels correspond to BT2949 numbering) and appears in close proximity to other likely substrate-coordinating residues. Mannobiose appears to occupy two distal subsites away from the proposed catalytic pair. Conserved residues from all enzyme models are shown overlaid. (*a*) was assembled using *PyMOL* v.1.6 (Schrödinger). Conservation mapping in (*b*) was conducted using *Homolmapper* (Rockwell & Lagarias, 2007 ▶) using the native structure of BT2949 and the sequence alignment in Fig. 4 ▶(*a*) as inputs. (*b*) and (*c*) were assembled using *CCP*4*mg* (McNicholas *et al.*, 2011 ▶).

The tertiary structure of BT2949 comprises a single-domain protein adopting a classical (α/α)_6_-barrel fold (Fig. 3 ▶ *a*). Despite low sequence similarity (BT2949 shares only 16–25% identity with other GH76s from *Bt* and a maximum of 30% across the family as a whole), the overall structure of BT2949 appears well conserved with other currently known GH76 enzymes [PDB entries 3k7x (*Listeria innocua*; Northeast Structural Genomics Consortium, unpublished work), 4c1s (*Bt*; Cuskin *et al.*, 2015 ▶), 4mu9 (*Bt*; Joint Center for Structural Genomics, unpublished work) and 4bok (*Bacillus circulans*; A. Striebeck, V. S. Borodkin, A. T. Ferenbach & D. M. F. van Aalten, unpublished work)]. Indeed, a structural comparison of BT2949 and the four known family members using the *DALI* server (Holm & Rosenström, 2010 ▶) produced respective *Z*-scores of 37.4, 43.9, 43.1 and 34.2 and r.m.s.d. values of 2.2, 2.4, 2.1 and 2.9 Å mapped across 325, 346, 334 and 314 C^α^ positions in each case. Thus, despite widely varying primary amino-acid sequences, possibly indicating distant evolutionary backgrounds, the observation of a common structural motif, particularly among the other *Bt* enzymes, strongly implies conserved functionality/activity between BT2949 and other proteins belonging to the GH76 family.

Unusually, considering the large and highly branched nature of α-mannan substrates, the BT2949 barrel motif appears relatively undecorated, with only four major loop structures, comprising several short β-strands, linking consecutive helices on one face of the molecule (see Fig. 3 ▶ *a*). Together with the natural cavity at the centre of the barrel structure, this loop-adorned region forms a large and highly negatively charged cleft consistent with a site adapted for binding extended carbohydrate moieties (Davies & Henrissat, 1995 ▶). Assignment of potential catalytic function is further reinforced by the observation that much of the relatively small degree of sequence conservation is maintained within this extended pocket (Fig. 3 ▶ *b*). Typical glycosidase active sites comprise a carboxylate pair as the principal catalytic residues and are often augmented by a variety of hydrogen-bond donors and aromatic side chains, forming π-stacking inter­actions, to assist with substrate coordination (Nerinckx *et al.*, 2003 ▶; Vocadlo & Davies, 2008 ▶). Within the proposed BT2949 active pocket, several solvent-exposed and well conserved (within *Bt* GH76 enzymes) carboxylates, Asp143, Glu144 and Asp249, can be observed in close proximity to several equally strongly conserved aromatic residues, Trp82, Trp147, Tyr265 and Phe315 (Figs. 3 ▶ *c* and 4 ▶ *a*). Furthermore, structural overlays with the previously solved *Bt* GH76 enzyme BT3782 (PDB entry 4mu9) reveals a fortuitously bound HEPES [4-(2-hydroxy­ethyl)-1-piperazineethanesulfonic acid] molecule, which is likely to be derived from the crystallization conditions, within 3 Å of Asp143 (Fig. 3 ▶ *c*). A not uncommonly observed ligand within various glycoside hydrolases, HEPES contains a six-membered piperazine ring that may well be capable of mimicking a carbo­hydrate and which here interestingly appears distorted towards a higher-energy boat conformation through its interaction with Asp143 and a coordinated water molecule. Together, these observations are strongly suggestive as to the location of the BT2949 active site, with Asp143 portrayed as a likely catalytic amino acid.

**Figure 4:**
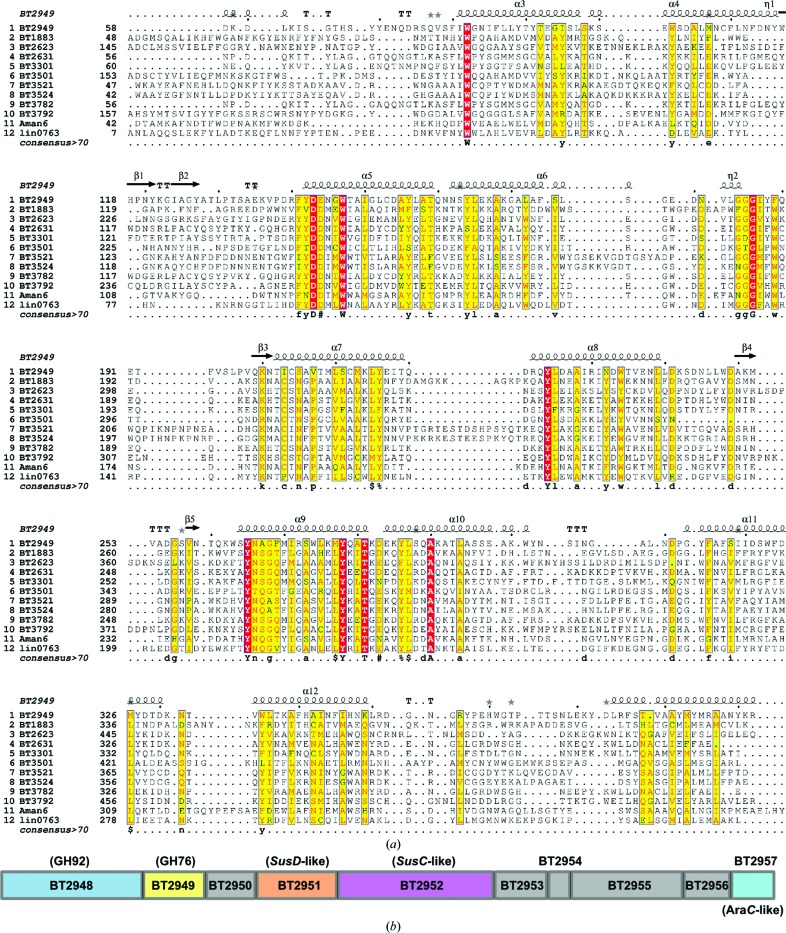
(*a*) Sequence alignment of the putative catalytic regions of the ten GH76 enzymes encoded by *Bt*, plus *Bc*GH76 and lin0763 from *L. innocua* (the remaining GH76 enzymes for which structures are currently known). Identical residues are shown highlighted in red, whilst highly similar amino acids are highlighted in yellow. Within the consensus sequence (bottom row), full identity is depicted by uppercase letters, a consensus score of >0.5 is depicted by lowercase letters and amino acids with similar chemical properties are indicated *via* symbols (! is I or V, $ is L or M, % is F or Y and # is any one of N, D, Q or E). (*b*) Organization of genes within PUL 43 (as defined by Martens *et al.*, 2008 ▶). Boxes are scaled relative to the size of the largest gene (BT2952, 2967 base pairs, 988 amino acids), with genes with known/assigned function shown in colour. The alignment in (*a*) was prepared using the *PRALINE* server (Heringa, 1999 ▶; Simossis & Heringa, 2005 ▶), with secondary-structure assignment using *ESPript* (Robert & Gouet, 2014 ▶). The three-dimensional structures of all known GH76 enzymes were included to inform the final alignment; however, for simplicity, only the secondary structure of BT2949 (the subject of this work) is shown.

Whilst Asp143 is fully conserved across all *Bt* GH76 enzymes (see Fig. 4 ▶ *a*), the neighbouring residue, Glu144, appears as an Asp in all other *Bt* proteins. Despite this apparent disparity, the reason for which remains unclear, we believe that these consecutive carboxylates are still highly likely to constitute the GH76 catalytic pair. Comparison of BT2949 with a newly released structure of the GH76 α-mannanase from *B. circulans* (*Bc*GH76, also referred to as Aman6) in complex with α-1,6-mannobiose (PDB entry 4boj; A. Striebeck, V. S. Borodkin, A. T. Ferenbach & D. M. F. van Aalten, unpublished work) reveals that, whilst highly conserved, the other potential catalytic amino acid, Asp249 (BT2949 numbering), appears to play a pre­dominant role as an assistant in substrate binding. In the *Bc*GH76 complex structure, mannobiose appears distal from the position equivalent to Asp143 and Glu144 (Asp124 and Asp125 in *Bc*GH76) and in close proximity to Asp249 (Asp228 in *Bc*GH76; see Fig. 3 ▶ *c*). Whilst the carboxylate group of the Asp249 (or equivalent) position does appear to point towards the glycosidic bond, somewhat reminiscent of catalytic acid or acid/base functionality, at a distance of approximately 4.8 Å and with no coordinating water molecules visible, a direct interaction between Asp249 and the ligand is highly unlikely. Most importantly, however, the anomeric position of the nonreducing mannoside appears to point directly out into solvent, leaving no possibility of enzyme-mediated nucleophilic attack (Fig. 3 ▶ *c*). As such, it is postulated that, whilst informative, the deposited *Bc*GH76 complex with α-1,6-mannobiose is more likely to be representative of the coordination of mannoside moieties in distal (−3 and −2) subsites rather than a genuine enzyme–substrate complex. This observation is supported by earlier findings showing that mannobiose is not a substrate of *Bc*GH76, with enzymatic activity increasing significantly with each additional mannoside added (Nakajima *et al.*, 1976 ▶).

Despite observations supportive of the role of BT2949 in hydrolysing α-mannans, like much of GH76 little or no detail is currently known about its specific activity. An understandable reasoning for this lies in the lack of available substrates for use in transcriptomic and/or biochemical studies (homogenous oligosaccharides are difficult to purify from source and, given their size, are equally tricky to synthesize). The *Bt* genome encodes ten enzymes classified into GH76, hinting at a large redundancy of general α-mannan-degrading activities. Detailed genomic and transcriptomic analyses (Xu *et al.*, 2007 ▶; Martens *et al.*, 2008 ▶), in addition to large-scale biochemical characterization of *Bt* GH92 α-mannosidases (Zhu *et al.*, 2010 ▶), has suggested that this expansion of enzymes, together with their respective PULs, functions as a series of ‘molecular toolboxes’. Individual PULs are capable of encoding for precise and subtly differing activities, which can be selectively upregulated in response to specific environmental cues such as the detection of glycans from a particular bacterial or fungal species. Thus, preliminary data indicating the specific glycan/mannan architectures sensed by response elements governing activation of individual PULs, and therefore the likely substrate specificity of individual enzymes, can be obtained through RT-PCR analysis of the organism cultured in the presence different carbohydrates as the primary carbon source. Such an approach is exemplified by recent work showing that certain *Bt* GH76 enzymes, and their respective PULs, are specifically upregulated by *S. cerevisiae* α-mannan, functioning to catalyse its complete degradation (Cuskin *et al.*, 2015 ▶). Despite extensive analysis against different glycans that contain an α-1,6-mannose backbone with an array of different side chains, however, no detectable activity/specificity has as yet been elicited for the BT2949-containing PUL, indicating that this particular enzyme is likely to function against as yet unknown or untested glycocon­jugates (Cuskin *et al.*, 2015 ▶). BT2949 is inactive against representative substrates from *S. cerevisiae* cell-wall α-mannan, including various linear α-1,6-mannooligosaccharides (lengths ranging from Man_2_ to Man_8_), and against common side-chain linkages including α-1,2-mannobiose and α-1,3-mannobiose.

As discussed above, much of the extensive carbohydrate-processing apparatus encoded by *Bt* is encoded by PULs where respective enzymes function as a complete unit, sensing and then catalysing both the specific breakdown and cross-membrane transport of a wide variety of natural glycoproducts (Martens *et al.*, 2008 ▶, 2009 ▶). The release of carbohydrate moieties from complex dietary polysaccharides, as well as the modulation of host N- and O-glycans lining the intestinal lumen, orchestrated by *Bt* PULs contribute significantly to both the nutrition and the general health of host organisms and as such makes these fascinating gene clusters highly worthy of study. To date, *Bt* is thought to encode a total of 88 PULs, 23 of which have been shown to be induced by mucin O-glycans, two by glycosaminoglycans (GAGs), one by starch and, interestingly, three by host N-glycans (Martens *et al.*, 2008 ▶). Most relevant, however, a further three PULs (BT2620–BT2632, BT3775–BT3779 and BT3787–BT3792) have been shown to be strongly upregulated when cultured in the presence of α-mannan from *S. cerevisiae* (Martens *et al.*, 2008 ▶), demonstrating a consistent role in targeting and hydrolysing α-mannoside-based polysaccharides present within the gut environment.

Regardless of their polysaccharide targets, all *Bt* PULs contain several common features, including homologues of *susC* and *susD* (the respective membrane-bound transport and binding proteins from the starch-utilization system), sensing regulators governing transcription, including σ/anti-σ factor pairs and hybrid two-component phosphorelay systems, and widely varying carbohydrate-modifying enzymes such as GHs, GTs, polysaccharide lyases (PLs) and sulfatases (Martens *et al.*, 2009 ▶). BT2949 is located in PUL 43, a relatively short locus that includes ten genes spanning BT2948–BT2957 (see Fig. 4 ▶ *b*). Despite the exact biological substrate of PUL 43 being as yet unknown, we can infer likely function against α-mannans/α-mannose-containing polysaccharides from the known activities and/or homologies of its component proteins. Immediately adjacent to BT2949 is BT2948, a characterized GH92 α-mannosidase with demonstrated activity against yeast α-mannan, whilst BT2950 shows similarity to the concanavalin A lectin family, a group of binding proteins known to target terminal α-mannosyl moieties (Fig. 4 ▶ *b*). The remainder of PUL 43 is made up of BT2951 and BT2952, which are the respective *susD* and *susC*-like membrane proteins, BT2953, another proposed membrane protein with similarity to the *rhaT* family of rhamnose transporters, and BT2954–BT2956, showing similarity to cupin, formate acetyl transferase and pyruvate-formate lyase family proteins, respectively. The final member of the locus, BT2957, is *AraC*-like and has been proposed to be a helix–turn–helix-type transcriptional regulator controlling transcription of the rest of the genes in the PUL (Xu & Gordon, 2003 ▶; see Fig. 4 ▶ *b*). Together, these observations strongly portray BT2949, and PUL43 as a whole, as a mannan-hydrolysing unit with likely activity against an as yet unknown or untested glycan architecture. Indeed, various species within the Sodariomycetes class of fungi, a large phylogenetic subdivision whose members function as leaf-litter decomposers, as well as being fungal parasites of both humans and insects, have been shown to produce surface heteromannans composed of mannoside, galactoside and rhamnoside sugars all linked to an extended α-1,6-mannan core. Furthermore, the side chains of *Schizosaccharomyces pombe* mannan are known to contain α-linked galactose moieties with pyruvate modifications at O1 and O3, potentially accounting for the proposed pyruvate lyase activity attributed to BT2956. Less commonly observed mannan architectures such as these might well act as substrates for PULs with currently unknown function and would fit well with the role of *Bt* as a ‘glycan specialist’ and in its general policing of the composition of the microbiota.

In summary, the role in human health played by dominant members of our gastrointestinal ecosystem such as *Bt* is of unquestionable importance, particularly in light of the rapidly evolving nature of the modern human diet. With greater access to more varied produce and foodstuffs than ever before, and the growing problems of obesity and type II diabetes in society, it is perhaps more vital than ever to understand how the behaviour and composition of our internal microbial communities affects our own metabolism and how external factors can be used to influence this relationship. Detailed metabolomic, transcriptomic and biochemical characterization of the organisms, gene clusters and individual enzymes involved in the breakdown and modification of polysaccharides present within the gut lumen will undoubtedly further this objective. The structure of BT2949 presented here will augment the relatively limited data that are available on GH76 enzymes and will aid in the future characterization of α-mannan hydrolysis by *Bt*.

## Supplementary Material

PDB reference: BT2949, 4v1s

PDB reference: selenomethionine derivative, 4v1r

## Figures and Tables

**Table 1:** Data-processing, phasing and refinement statistics for BT2949 Values in parentheses are for the highest resolution shell

	Selenomethionine derivative	Native
Data processing		
Space group	*P*22_1_2_1_	*P*22_1_2_1_
Unit-cell parameters ()	*a* = 81.7, *b* = 120.7, *c* = 126.2	*a* = 81.7, *b* = 121.0, *c* = 125.6
Wavelength ()	0.97926	0.97630
Molecules in asymmetric unit	2	2
Resolution range ()	48.551.80 (1.831.80)	46.041.50 (1.531.50)
*R*_merge_ †	0.075 (0.441)	0.081 (0.679)
*I*/(*I*)	14.2 (3.4)	11.0 (1.8)
CC_1/2_	0.94	0.827
Completeness (%)	99.9 (99.9)	90.3 (51.8)
Multiplicity	7.9 (6.7)	6.7 (4.3)
Phasing		
Anomalous completeness (%)	99.6 (99.5)	
Anomalous multiplicity	4.0 (3.3)	
CC_anom_	0.676 (0.086)	
No. of sites (occupancy 0.5)	17	
FOM (2.5)	0.76	
Refinement		
No. of reflections (unique)	110076	170173
No. of atoms used in refinement	7009	7016
*R*_cryst_ (%)	14.8	14.9
*R*_free_ (%)	16.8	18.0
Mean *B* values (^2^)		
Protein	20.8	18.5
Solvent	40.4	42.5
R.m.s.d., bonds ()	0.011	0.011
R.m.s.d., angles ()	1.38	1.35
Ramachandran statistics (%)		
Preferred	98.3	98.6
Allowed	1.5	0.9
Outliers	0.3	0.6
PDB code	4v1r	4v1s

† The formulae for *R*_merge_ and *R* (*R*_cryst_ and *R*_free_), as applied within *AIMLESS* and *REFMAC*, are as follows: 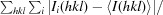 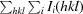, *R* = 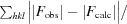 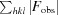.
